# Serum Antibody Levels to the *Pneumocystis jirovecii* Major Surface Glycoprotein in the Diagnosis of *P. jirovecii* Pneumonia in HIV+ Patients

**DOI:** 10.1371/journal.pone.0014259

**Published:** 2010-12-09

**Authors:** Kpandja Djawe, Laurence Huang, Kieran R. Daly, Linda Levin, Judy Koch, Alexandra Schwartzman, Serena Fong, Brenna Roth, Anuradha Subramanian, Katherine Grieco, Leah Jarlsberg, Peter D. Walzer

**Affiliations:** 1 Veterans Affairs Medical Center, Cincinnati, Ohio, United States of America; 2 Division of Epidemiology and Biostatistics, Department of Environmental Health, University College of Medicine, Cincinnati, Ohio, United States of America; 3 Division of Pulmonary and Critical Care Medicine and HIV/AIDS Division, San Francisco General Hospital, University of California San Francisco, San Francisco, California, United States of America; 4 Division of Infectious Diseases, Department of Internal Medicine, University of Cincinnati College of Medicine, Cincinnati, Ohio, United States of America; Albert Einstein College of Medicine, United States of America

## Abstract

**Background:**

*Pneumocystis jirovecii* remains an important cause of fatal pneumonia (*Pneumocystis* pneumonia or PcP) in HIV+ patients and other immunocompromised hosts. Despite many previous attempts, a clinically useful serologic test for *P. jirovecii* infection has never been developed.

**Methods/Principal Findings:**

We analyzed serum antibody responses to the *P. jirovecii* major surface glycoprotein recombinant fragment C1 (MsgC1) in 110 HIV+ patients with active PcP (cases) and 63 HIV+ patients with pneumonia due to other causes (controls) by an enzyme-linked immunosorbent assay (ELISA). The cases had significantly higher IgG and IgM antibody levels to MsgC1 than the controls at hospital admission (week 0) and intervals up to at least 1 month thereafter. The sensitivity, specificity and positive predictive value (PPV) of IgG antibody levels increased from 57.2%, 61.7% and 71.5% at week 0 to 63.4%, 100%, and 100%, respectively, at weeks 3–4. The sensitivity, specificity and PPV of IgM antibody levels rose from 59.7%, 61.3%, and 79.3% at week 0 to 74.6%, 73.7%, and 89.8%, respectively, at weeks 3–4. Multivariate analysis revealed that a diagnosis of PcP was the only independent predictor of high IgG and IgM antibody levels to MsgC1. A high LDH level, a nonspecific marker of lung damage, was an independent predictor of low IgG antibody levels to MsgC1.

**Conclusions/Significance:**

The results suggest that the ELISA shows promise as an aid to the diagnosis of PCP in situations where diagnostic procedures cannot be performed. Further studies in other patient populations are needed to better define the usefulness of this serologic test.

## Introduction

*Pneumocystis jirovecii* pneumonia (PcP) was the leading cause of morbidity and mortality in HIV+ patients early in the HIV/AIDS epidemic [1]–[3]. With the introduction and wide use of highly active antiretroviral therapy (ART) and PcP chemoprophylaxis, the incidence of PcP in this patient population has declined. However, PcP remains an important clinical problem in HIV+ and other immunocompromised patients with mortality rates ranging from 10–60% depending on the underlying disease [2]–[3].

Definitive diagnosis of PcP is usually made by the microscopic demonstration of the organism in specimens obtained by induced sputum or bronchoalveolar lavage fluid (BALF) with histological or immunofluorescent reagents [4]. Often, HIV+ patients with a suggestive clinical picture of PcP are treated empirically for PcP [5]. In such cases, non-invasive and non-specific methods such as chest radiographs, serum lactic dehydrogenase (LDH), or serum β-glucan levels may be used to help support the diagnosis [6]–[9]. Detection of *P. jirovecii* DNA by polymerase chain reaction (PCR) is highly sensitive; however, this test is not commercially available, and the high rate of *P. jirovecii* colonization in HIV+ patients can make PCR results difficult to interpret [10].

The availability of a reliable and sensitive serological test for *P. jirovecii* infection, particularly if it involved only a single specimen, is attractive because it would provide a degree of specificity to currently available non-invasive tests described above. Serologic studies have been investigated for many years, but the reagents used could not reliably distinguish present from past *P. jirovecii* infection or colonization from active disease [11]–[19]. Since *Pneumocystis* cannot be reliably grown in vitro, it has been difficult to obtain large quantities of purified proteins for use as antigens for assay development.

Recently, recombinant antigens of *P. jirovecii* have been developed that show promise as reagents for serologic studies: Kexin 1, which is encoded by a single gene; the major surface glycoprotein (Msg), which is encoded by multiple genes and is capable of antigenic variation [20]–[21]. Both antigens are highly immunogenic and contain protective epitopes [19]–[23]. We have focused our attention on Msg. First we developed 3 overlapping recombinant fragments (MsgA, MsgB, MsgC1) that span the entire length of a single Msg isoform for our studies [24]–[25]. Then we developed variants (MsgC 3, 8, and 9) of Msg C1 in order to better define the reactivity of serum antibodies [26].

We have shown that MsgC1 is helpful in distinguishing HIV+ patients who have had previous PcP from those who did not and in differentiating healthcare workers who had contact with patients from those who did not [24]–[25], [27]. We also examined the serologic responses to *P. jirovecii* infection in early childhood; geographic differences in seroreactivity to MsgC1; and the specific factors independently related to high antibody levels to MsgC1 in long-term cohort study [28]–[31]. In addition, we conducted a pilot study to examine the serologic responses in HIV+ patients admitted to the San Francisco General Hospital (SFGH) with PcP (cases) and pneumonia due other causes (controls) [32]. The data showed that first episode of PcP and CD4+ counts ≥50 cells/µL were the principal host factors associated with a rise in antibody response to MsgC1. While this report was of interest, it was limited by the relatively small number of cases and lack of convalescent serum specimens in the controls. In addition, the study only examined IgG antibodies and provided only modest information about the influence host factors had on these antibodies.

Since the pilot study, we have increased the number of SFGH HIV+ cases and controls with acute and convalescent serum specimens. We have also improved the IgG assay and developed an assay to measure IgM antibody responses to MsgC1. We undertook the present study to: 1) characterize the IgG and IgM antibody responses to MsgC1 in the cases and the controls; 2) evaluate these antibody responses in the diagnosis of PcP; and 3) identify specific host or environmental factors associated with these antibodies. The data show that there are significant differences in IgG and IgM levels between cases and controls at multiple time points, which suggest that this serologic test may be helpful in the diagnosis of PcP.

## Materials and Methods

### Patient population

The study was approved by the University of California San Francisco and the University of Cincinnati Institutional Review Boards, and all patients provided written, informed consent for participation in the study. As standard clinical practice, all HIV+ patients admitted to SFGH with respiratory symptoms were evaluated by a uniform diagnostic protocol previously described [33]. The diagnosis of PcP was confirmed by visualization of the organism in induced sputum or bronchoalveolar lavage fluid (BALF) specimen (cases) using Diff-Quik stain, which is a modified Giemsa stain, which identifies all stages of the *Pneumocystis* life cycle. Patients whose specimens were negative for *P. jirovecii* served as controls. From May 2000 to May 2008, 173 HIV+ subjects (110 cases, 63 controls) were enrolled. Among the 63 controls, 45 (71.4%) had community acquired pneumonia (CAP), 7 (11.1%) had mycobacterial diseases (2 with tuberculosis and 5 with *Mycobacterium avium* complex (MAC)), and 7 (11.1%) had other diseases such as pulmonary Kaposi sarcoma, bronchitis, pulmonary fibrosis, and chronic obstructive pulmonary disease (COPD). Four patients had no discharge diagnosis listed in chart abstraction. Seven of the patients with CAP had bacteria cultured in sputum and 2 had MAC isolated in sputum.

### Data collection

At hospital admission, clinical and demographic (age, gender, race) characteristics were collected [32]–[33]. The clinical data collected were history of PcP, CD4+ counts, plasma HIV viral load, serum LDH level, serum albumin levels, arterial blood gas measurements and the use of mechanical ventilation. The use of PcP prophylaxis within the last 3 months and PcP treatment provided were also collected. An acute-phase serum specimen was drawn at the time of hospital admission and convalescent-phase serum specimens were drawn every 1–2 weeks for 6 weeks. Serum specimens were stored at −80°C and shipped to the University of Cincinnati and the Cincinnati VA Medial Center for analysis.

### Recombinant antigens

The recombinant MsgC1 fragment was prepared by PCR using DNA isolated from *P. jirovecii* infected lung or cloned *Msg* genes as templates and Amplitaq enzyme (Applied Biosystems, Foster City, CA) to generate *msg* gene segments [20], [24]–[25]. The PCR products were cloned into the pET30 vector (Novagen, Madison WI) and the recombinant MsgC1 proteins were expressed in *Escherichia coli* and purified as previously described [24].

### IgG ELISA

The ELISA was performed as previously described [25]–[26], [31]–[32]. Serum specimens to be analyzed and the standard reference serum were tested against recombinant MsgC1; PBS without antigen was used as a negative control. The reactivity of each serum specimen to Msg was corrected by subtraction of the reactivity of that serum to PBS (mean OD Msg – mean OD PBS), and the results were quantified using the method of Bishop and Kovacs [34]. A standard serum with specificity for MsgC1 was generated by mixing a pool of 4–6 sera with high reactivity to MsgC1. A standard curve was generated for MsgC1 on each day of the assay and was used to calculate the units of reactivity to MsgC1. The standard serum pool was assigned a value of 100U of reactivity to MsgC1 construct in 100 µL of a 1: 100 dilution of serum. Sera were assayed at 1: 100 to 1: 200 dilutions to fit the linear portion of the standard curves and units of reactivity were calculated taking the dilution into account.

### IgM ELISA

The IgM antibody reactivity to MsgC1 was measured using the same ELISA methods described above for IgG antibody reactivity except for the following changes. The secondary antibody was affinity-purified, HRP-labeled goat anti-human IgM (μ chain) (KPL Products, Gaithersberg, MD) instead of goat anti-human IgG (H&L) (KPL Products) was used for IgG antibodies. In a previous study, we tested the anti-IgM reagent for μ chain specificity in two ways: 1) with serum from HIV+ patients with heavy staining IgM antibody bands to *P. jirovecii* that had been separated by column chromatography into IgM and IgG fractions; and 2) with purified IgM and IgG on immunoblots [11]. In both cases, anti-IgM antibody only reacted with the IgM preparations, indicating that it was μ chain-specific. As an additional measure of specificity, we have recently screened 200 healthy blood donors for IgM and IgG using the same IgG and IgM ELISA. Of 200 serum samples examined, IgG antibodies were detected in 103 (51.5%) of the specimens, whereas IgM antibodies were only detected in 7 (3.5%) of the specimens (p<0.001).

### Statistical Analysis

Descriptive statistics were calculated to describe patient clinical and demographic characteristics. Categorical variables were analyzed by chi square statistics. Bivariate correlations measuring associations between MsgC1 antibodies and clinical variables at initial visit, and the auto-correlation between MsgC1 antibody responses on the same patient were calculated. The relationship of PcP infection to MsgC1 antibody levels was determined by the parameter estimate of dichotomized PcP infection in a linear mixed model, applied for modeling correlated log_e_ MsgC1 antibody levels with left censoring. Mixed models are often used for estimating fixed effects and components of variability in longitudinal studies of continuous data. The model included a random intercept, which defined MsgC1 antibody values on the same patient to be correlated. Previous episode of PcP, CD4+ count (<50, ≥50 cells/µL), and one additional clinical variable, were modeled as fixed effects, in addition to PcP. Analyses were repeated when the additional clinical variable was replaced by a different clinical variable and all other variables remained the same. MsgC1 antibody responses during the first 6 weeks of follow-up were analyzed. For each interval, sensitivity and specificity were calculated for predicting PcP status, when MsgC1 antibody levels were dichotomized as above or below the cut points. We used parametric receiver operating characteristic (ROC) curve analysis methods to generate different cutpoints in each interval. The optimum cutpoint which was the same as the median value in each time interval was used to calculate the sensitivity and the specificity. SAS for Windows, version 9.2 (SAS Institute, Cary, NC) was used for all analyses and the statistical significance was based on the 5% level. The graphs were produced using R software, version 2.9 (R project for statistical computing).

## Results

### Demographic characteristics

At hospital admission, there were statistically significant differences in gender and racial distributions between PcP cases and controls **(Table S1)**. The cases were more likely to be males (88.2% vs. 74.6%) (p = 0.02) and Caucasians (52.7% vs. 33.3%) (p = 0.046).

### Clinical characteristics

There was no statistical difference in the proportion of patients with prior episodes of PcP between cases and controls (16% and 23.8% respectively) **(Table S1)**. However, cases had a more advanced stage of HIV illness with significantly higher median HIV viral load (1.3×10^5^ vs. 3.6×10^4^ copies/mL) (p<0.01) and a higher proportion of patients with CD4+ counts <50 cells/µl (62.4% vs. 34.9%) (p<0.01) than controls. Pneumonia severity was similar between cases and controls. Both groups had similar serum albumin levels, arterial blood gas measurements, and were unlikely to require mechanical ventilation. However, cases had a significantly higher median LDH level (347 U/L) compared to controls (283 U/L) (p<0.01). Fewer cases (20.0%) than controls (39.7%) were taking PcP prophylaxis within 3 months before hospital admission (p<0.05). All cases (100%) were treated for PcP while undergoing diagnostic evaluation compared to 66.7% of the controls (p<0.01), and the cases were more likely to be treated with trimethoprim-sulfamethoxazole (TMP-SMX) compared to controls (74.6% vs. 55.6%) (p<0.01). A significant proportion of cases (82.7%) were on corticosteroids compared to controls (41.3%) (p<0.01).

### IgG and IgM antibody levels to MsgC1 at admission and over time

**Figure 1:**
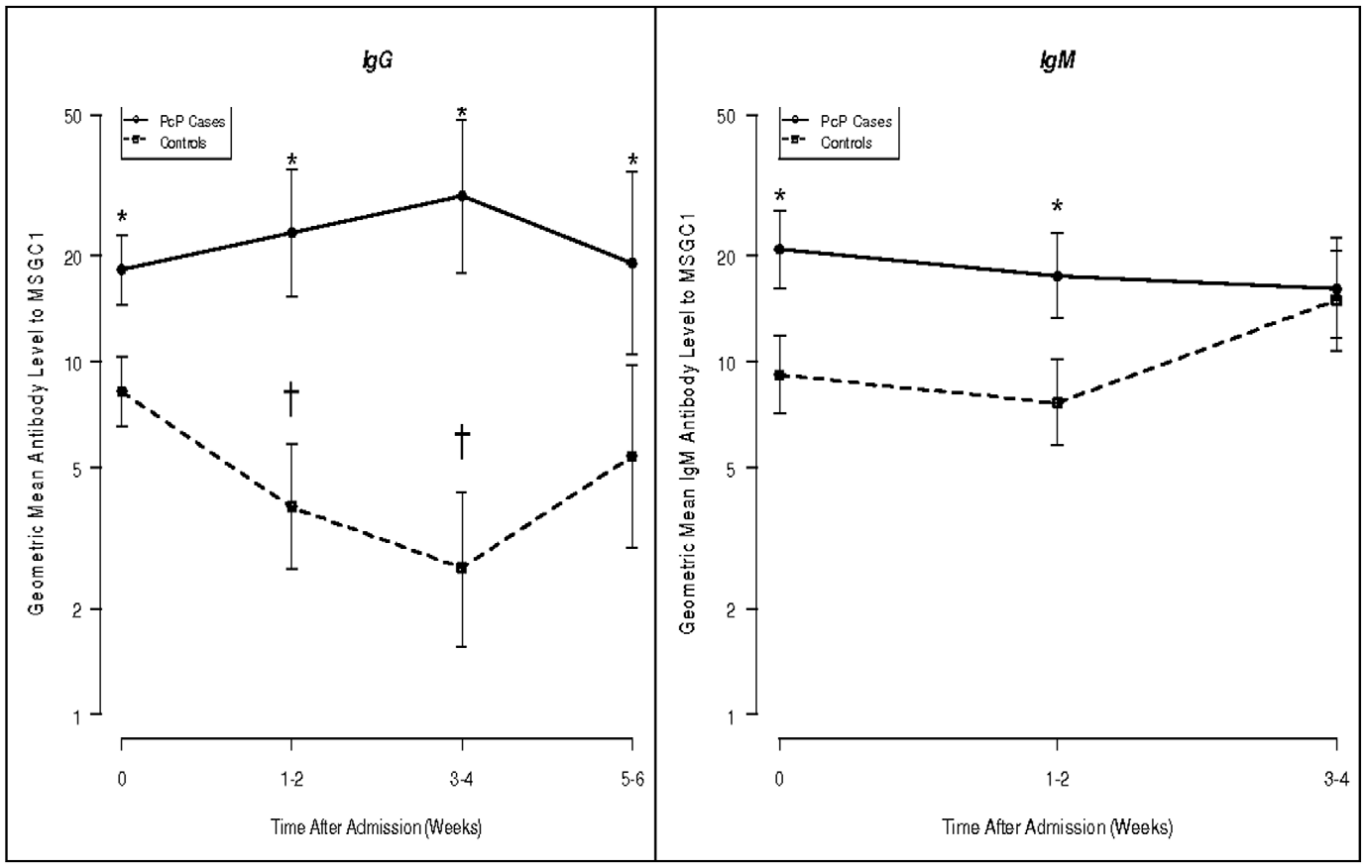
Geometric mean [95% CI] IgG and IgM antibody levels to MsgC1 in cases and controls over time. *p<0.05 tests the differences in mean values between cases and controls at each time point. † tests in controls only the differences in mean values at week 1–2 compared to baseline and at week 3–4 compared to baseline. These comparisons in cases were not significant. A mixed effect Tobit regression model was written which provided an estimate of the average effect of PcP+ compared to PcP− in each interval. A random effect subject effect was modeled to account for correlation between measurements on the same subject in the same interval. The value of the t-statistics, testing Ho: PcP parameter estimate  = 0, was used to judge the statistical significance of the effect of PcP+, compared to PcP−.

We compared changes in mean IgG antibody levels to MsgC1 for a period of up to 6 weeks, and changes in mean IgM antibody levels to MsgC1 for a period of up to 4 weeks between PcP cases and controls **(Table S1,** **Figure 1** **)**. IgG antibody levels were significantly higher in the cases than in the controls at all time points (p<0.05). The IgG antibody levels in the cases increased from 18.15 (95% CI: [14.46–22.78]) at admission to their peak of 29.30 (95% CI: [17.73–48.42]) at weeks 3–4 and then declined to 18.95 (95% CI: [10.45–34.37]) at weeks 5–6. However, these changes were not statistically significant. The IgG antibody levels for the controls declined from 8.17 (95% CI: [6.55–10.28]) at admission to 2.59 (95% CI: [1.56–4.27]) at weeks 3–4 and then rose slightly to 5.38 (95% CI: [2.97–9.77]) at weeks 5–6. The changes in the controls were statistically significant in the first four weeks of the study (p<0.05).

IgM antibody levels in the cases were significantly higher than for the controls at the time of hospital admission and at weeks 1–2 thereafter (p<0.05) (**Table S1,** **Figure 1** **)**. IgM antibody levels in the cases were highest at baseline (20.73) and then decreased slightly over time. Antibody levels in the controls were 9.16 at baseline, then declined at weeks 1–2 and rose at weeks 3–4.

When serum specimens for the entire 6 weeks period were combined, PcP cases still had significantly higher mean IgG antibody levels than controls (mean [95%CI]:17.39 [14.50–20.86] vs. 7.40 [6.17–8.88]) (p<0.05). Similarly, the combined mean IgM antibody level to MsgC1 in the cases was higher than that in the controls (mean [95% CI]: 21.63 [18.69–25.02] vs. 11.51 [9.95–13.31]) (p<.05). By paired analysis of baseline serum specimens and 3–4 week specimens, 46% of the cases developed a median rise of 1.74-fold in IgG antibody levels at weeks 3–4 compared to 14% of the controls with a median rise of 1.03-fold. However, the difference in proportions was not statistically significant.

**Figure 2:**
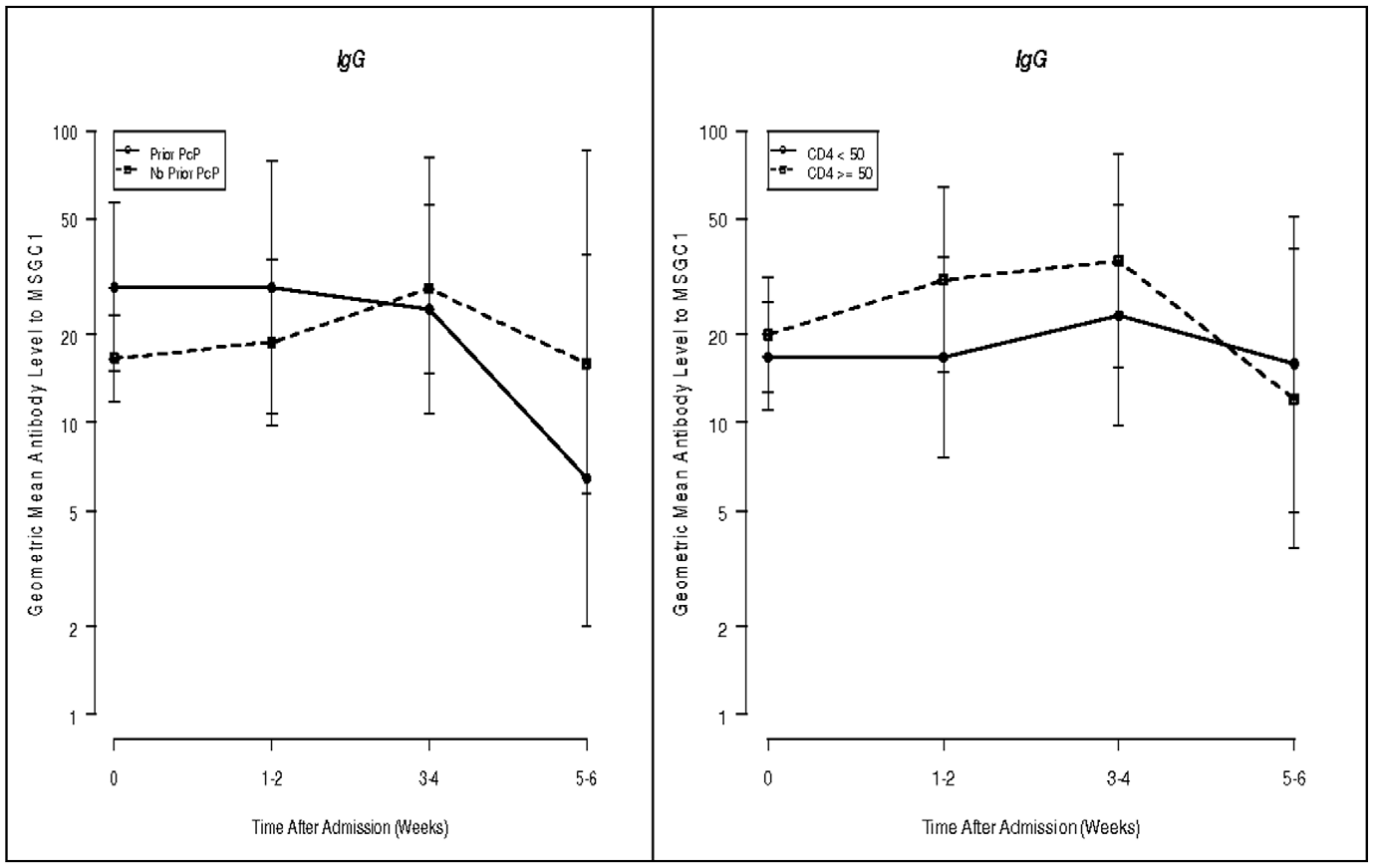
Geometric mean [95% CI] IgG antibody levels to MsgC1 in cases by history of PcP and CD4+ cell counts over time.

We attempted to identify independent risk factors associated with a rise in IgG antibody levels to MsgC1 among PcP cases at weeks 3–4. In light of data in our pilot study, we were particularly interested in the role of first episode of PcP and CD4+ counts. As seen in **Figure 2**, there were no significant differences in the sequential antibody levels to MsgC1 between first episodes and repeated episodes of PcP. Subjects with CD4+ counts ≥50 cells/µL had consistently higher antibody levels during weeks 1–4 than subjects with CD4+ counts <50 cells/µL, but these differences never reached statistical significance.

### Use of antibodies to MsgC1 in the diagnosis of PcP

For analysis using IgG and IgM antibody levels to predict PcP diagnosis, cutpoint values were determined for different time points. A cutpoint value was defined to be the median value of all serum specimens from both cases and controls at a given time point. The IgG cutpoint values increased from baseline to weeks 3–4 and declined afterward **(** **Table 1** **)**. A similar pattern was observed in the sensitivity, specificity, and positive predictive value (PPV). At hospital admission, an IgG cutpoint of 23 was used to discriminate cases and controls with 57.2% sensitivity, 61.7% specificity and 71.5% PPV. By weeks 3–4, an IgG cutpoint of 40.5 was used to discriminate PcP cases and controls with 63.4% sensitivity, 100% specificity and 100% PPV. Thus, the increase in PPV over time was mainly due to an increase in specificity, which reflected changes in antibody levels both in the cases and controls.

**Table 1:** Sensitivity, Specificity, and Positive Predictive Value (PPV) Associated with IgG to MsgC1 Cutpoints at Each Time Interval Comparing PcP Cases and Controls.

Time	Samples in Cases	Samples in Controls	MsgC1 Cutpoint	Sensitivity	Specificity	PPV
Baseline	180	107	23	57.2	61.7	71.5
1–2 weeks	59	27	25	61.0	70.4	81.8
3–4 weeks	41	11	40.5	63.4	100	100
5–6 weeks	32	16	20.5	53.1	56.3	70.8

Note: We included 110 individual PcP cases and 63 individual controls in this study. Since some patients had their first specimen drawn at the time of hospital admission, but others had it drawn a day after admission, we defined baseline specimen to be specimen drawn on the day of hospital admission (day 0) or the following day after admission (day 1). Some subjects had specimens on both days and this resulted into 180 specimens for cases and 107 sample for controls.

In contrast to the IgG cutpoint which increased significantly over time, the IgM cutpoint decreased slightly over time **(** **Table 2** **)**. Sensitivity, specificity and PPV increased from 59.7%, 61.3%, and 79.3% at admission to 74.6%, 73.7%, and 89.8%, respectively, at weeks 3–4.

**Table 2:** Sensitivity, Specificity, and Positive Predictive Value (PPV) Associated with IgM to MsgC1 Cutpoints at Each Time Interval Comparing PcP Cases and Controls.

Time	Samples in Cases	Samples in Control	MsgC1 Cutpoint	Sensitivity	Specificity	PPV
Baseline	77	31	23.49	59.7	61.3	79.3
1–2 weeks	77	36	20.39	64.9	69.4	82.0
3–4 weeks	32	19	20.36	74.6	73.7	89.8

Note: We had 110 individual PcP cases and 63 individual controls in this study. Since some patients had their first specimen drawn at the time of hospital admission, but others had it drawn a day after admission, we defined baseline specimen to be specimen drawn on the day of hospital admission (day 0) or the following day after admission (day 1). Some subjects had specimens on both days and this resulted into 77 specimens for cases and 31 sample for controls. There are different numbers of specimens for IgG and IgM in each group because some subjects was not enough for both IgG and IgM antibodies to be tested.

### Factors associated with high IgG and/or IgM antibody levels to MsgC1

Here, we were interested in host factors associated with high antibody levels to MsgC1 rather than a rise or fall in antibody levels over time **(Table S2)**. In multivariate analysis using all IgG levels from both cases and controls as the main dependent variable, we found that PcP diagnosis and LDH levels were significant predictors of IgG antibody levels to MsgC1 when controlling for CD4+ count and previous episode of PcP. PcP diagnosis was significantly associated with high IgG antibody levels (p<0.01). In contrast, LDH levels had a negative effect on IgG antibody responses. One standard deviation increase in log_e_ LDH levels was significantly associated with decrease in IgG antibody levels (p = 0.02). When IgM was used as the main dependent variable, only PcP diagnosis was found to be a significant predictor of IgM antibody levels to MsgC1. PcP diagnosis was significantly associated with increased IgM antibody levels when controlling for previous episode of PcP and CD4+ count (p<0.05). LDH was associated with a decrease in IgM antibody levels, but the effect was not statistically significant. The effects of CD4+ counts, HIV viral load, PO_2_ levels and PcP prophylaxis on both IgM and IgG antibody levels were not statistically significant. The other variables such as race, gender, age, PcP treatment, albumin levels and the use of mechanical ventilation were modeled, but their effects on IgM and IgG antibody levels were not statistically significant (results not shown).

## Discussion

The present study analyzed acute and convalescent serum antibody responses to MsgC1 in HIV+ subjects with pneumonia due to *P. jirovecii* (cases) or other causes (controls) at a single site. The data showed that the cases had significantly higher IgG and IgM antibody levels than the controls both at the time of hospital admission and at intervals for up to one month thereafter. The sensitivity, specificity and PPV of these specimens increased from 57.2%, 61.7% and 71.5% at admission to 63.4%, 100%, and 100% at 3–4 weeks, respectively for IgG antibodies, and from 59.7%, 61.3%, and 79.3% at admission to 74.6%, 73.7%, and 89.8%, respectively for IgM antibodies. Multivariate analysis revealed that two factors were independently associated with altered antibody IgG and/or IgM antibody levels: the diagnosis of PcP was significantly associated with high IgG and IgM antibody levels; high LDH levels were associated with low IgG antibody levels.

The present report extends the results of our pilot study [32], but also revealed some differences. For example, the pilot study found no significant difference in antibody levels to MsgC1 between cases and controls at admission. Yet, that study did find significant differences in antibody levels between baseline and weeks 3–4; CD4+ counts <50 cells/µL and ≥50 cells/µL; and first episodes and recurrent episodes of PcP [32]. The present report found similar trends in these results, but they never reached statistical significance. Some reasons for these differences may involve a greater number of subjects in the present report; slight modifications in the ELISA in the last few years; and differences in statistical methods used to analyze the data. The analysis in the pilot study did not take into account values that were below the limit of detection; yet, in the present report we applied a regression model, which was able to handle censored values.

It is difficult to compare our study with previous reports because most of those studies used different antigen preparations, controls and methodology [11]–[19]. In these other studies, HIV+ patients with PcP exhibited serum antibody levels that were either similar to or lower than antibody levels of non-PcP patients at the time of diagnosis [11]–[15], [17]. The PcP patients also had a poor antibody response in convalescent specimens. Yet, studies of individual native *Pneumocystis* antigens suggested how these moieties might be useful in the future. In one report, technetium-labeled monoclonal antibodies to Msg showed promise (sensitivity of 85.7%, specificity of 86.7%) in using lung scans for the diagnosis of PcP [35]. Unfortunately, the test was never commercially developed. Another report that used native Msg as the antigen found that HIV+ patients with PcP had more frequent detectable antibodies and higher antibody levels than HIV+ patients without PcP and healthy controls [18]. On the other hand, a study that used a different recombinant Msg fragment than the one used in our study found high antibody levels in non-HIV immunocompromised patients who experienced PcP; however, there were no differences in antibody levels in HIV+ patients with PcP, HIV+ patients without PcP, or healthy controls [34].

One way that serology can be used in the diagnosis of infectious diseases is to demonstrate that the antibody level in a single serum specimen is above the established normal range of values. The attractiveness of this approach is that it avoids the need to obtain a second (convalescent) specimen. The sensitivity, specificity and PPV of the serologic data at the time of hospitalization in our study were not as good as we had hoped. The reasons for these findings are unclear but may involve several factors: the severity of HIV and immunosuppression in the cases; *P. jirovecii* colonization in the controls, which can be quite common [10]; previous episodes of PcP; and antibody cross-reactivity with other microbial antigens, although little is known about this subject. The fact that the cases consistently had higher antibody levels than the controls and that the specificity and PPV of the serologic test increased over time is encouraging. The decrease in antibody levels contributed to these changes, but the reasons for this decrease are unknown. It is also possible that the serologic test will be more sensitive in analyzing differences when used in non-HIV patients with PcP, as shown in the higher antibody levels to recombinant Msg in these patients [34].

The other way to use serology as a diagnostic tool is to demonstrate a rise in antibody levels over time. When serial dilutions are used to measure antibody levels, a 4-fold or greater rise is considered to be significant. Yet, in our ELISA, the determination of what constitutes a significant rise is more complicated because serum specimens are diluted only once to a specific level (e.g., 1/100) and the antibody level is then derived from a standard curve. In addition to the present study, several other reports have shown that about 40–50% of HIV patients with PcP develop a rise in antibodies to Msg [18], [31]–[32]. The increase in antibody levels in these patients helps support the diagnosis of PcP, but the failure to develop a rise (which occurred in the majority of cases) does not carry the same negative weight. The reasons for the relatively low frequency of antibody rises in PcP subjects are probably related to the advanced stage of HIV disease and its associated immunosuppression.

In light of the impaired B cell function in HIV infection and the low or absent IgM antibodies to *P. jirovecii* antigens in previous reports [13], [16], [36]–[37], the presence of elevated antibody levels in the present study is somewhat unusual and unexpected. However, we previously showed that HIV+ patients with single or recurrent episodes of PcP can develop active IgM and/or IgG responses to crude *P. carinii* antigens, and that the secondary anti-IgM antibody, which was essentially the same product as used here, was μ-specific [11]. In addition, IgM antibodies to specific antigens have been found in HIV+ patients with CD4+ >100 cells/µL who were immunized with *Haemophilus influenza*e Type b vaccine, and in the saliva of HIV+ patients with *Toxoplasma* encephalitis [38]–[39]. The elevated IgM antibodies in the present study might be in response to new infection, especially if this is the first episode of PcP. If this is a recurrent episode, there might be a different strain of *P. jirovecii* that would elicit this response. One study showed that some patients who experienced 2 episodes of PcP greater than 6 months apart tended to be infected with different *P. jirovecii* isolates rather than the same isolate [40]. Also, the IgM antibodies may have been elicited by Msg that underwent antigenic variation in an already acquired strain of *P. jirovecii*.

The high antibody levels to MsgC1 raise questions about the host and environmental factors that contribute to them. In a recent long-term study of HIV+ patients, we found that previous episode of PcP, geographic location, age and failure to take PcP prophylaxis were independently associated with increased IgG antibody levels to the recombinant MsgC1 fragments [31]. However, in the present study, we did not find significant effects of race, age, and gender on MsgC1 antibody levels. The discrepancy between this study and our previous report [31] about the effect of age on MsgC1 antibody levels might be due to the differences in study design. In that study, MsgC1 antibody levels were measured on average 6 months after the diagnosis of PcP, but in this study, it was measured within weeks. As in our pilot study [32], the cases and controls in the present report differed significantly in terms of their CD4+ counts, HIV viral loads, and LDH levels. Other investigators have also found higher LDH levels in PcP than in other forms of pneumonia [2], [4], [6]–[7]. Thus, despite having more advanced HIV disease and more severe lung damage, cases were still able to develop higher antibody levels than the controls. Cases in the present report were also significantly more likely to be male and Caucasian than controls, whereas in the pilot study these differences did not reach statistical significance [32]. The influence of race on PcP risk has shown conflicting results [31], [41]–[42]. Yet, with recent studies showing that PcP acquisition is influenced by host single nucleotide polymorphisms (SNPs) in specific genes [43]–[44], race might have a role in PcP incidence. Even though more studies are needed to elucidate this genetic association, the differences in PcP cases among racial groups found in this study might be due to genetic variation across different races.

This study has several limitations. It was only performed in HIV+ patients, who are generally more immunosuppressed than most other populations at risk for PcP. Thus, the results obtained here may underestimate the diagnostic value of this serologic test. Similarly, the population here was entirely composed of adults. The value of the study in pediatric HIV+ patients with PcP needs to be evaluated [45]. Since the study was conducted at a single center, the results may not reasonably apply to hospitals in different geographic locations. Also, only a minority of control subjects had an alternative, non-PcP diagnosis confirmed microscopically. While it is possible that these individuals may have had PcP and a false-negative Diff-Quik test, we have previously shown a high sensitivity (≥98%) and specificity (100%) of this assay at SFGH [33]. Thus, we believe that we have classified our cases and controls accurately.

In conclusion, this is the first large scale use of a recombinant *P. jirovecii* antigen in the serologic diagnosis of PcP in HIV+ patients. Overall, we are encouraged by the results and believe that they show promise as an aid to the diagnosis of PcP in situations where bronchoscopy cannot be performed. This promise may be increased by evaluating the test in different patient populations and in different geographic locations; delineating the areas of maximal reactions on Msg C1 and its closely related other Msg C fragments; and using Kexin 1 in combination with Msg C1. Serology may also be useful in conditions such as *P. jirovecii* colonization, as evidenced by decreased antibody levels to Kexin 1 [46] and by our finding of low antibody levels to Msg C fragments in smokers (Walzer, PD unpublished observations). Finally, the presence of serology as a tool to examine the risk of nosocomial transmission of *P. jirovecii* infections may be enhanced by the findings of increased antibody levels in hospital personnel with patient contact [27] and the presence of *P. jirovecii* in the air surrounding patients with PcP [47].

## Supporting Information

Table S1 Demographic characteristics and baseline clinical measurements of patients with *Pneumocystis jirovecii* pneumonia (PcP cases) and patients with pneumonia due to other causes (controls).
(0.04 MB DOC)

Table S2 The estimates and the associated p-value of the effects of different independent predictors on IgG and IgM antibody levels to MsgC1 in HIV-infected patients.
(0.04 MB DOC)
