# Supplementary material for: Serum Antibody Levels to the Pneumocystis jirovecii Major Surface Glycoprotein in the Diagnosis of P. jirovecii Pneumonia in HIV+ Patients
Source: PLoS One. 2010 Dec 9;5(12):e14259. doi: 10.1371/journal.pone.0014259 (PMC3000336; doi:10.1371/journal.pone.0014259)
Supplement: Table S1 — Demographic characteristics and baseline clinical measurements of patients with Pneumocystis jirovecii pneumonia (PcP cases) and patients with pneumonia due to other causes (controls). (0.04 MB DOC) [file pone.0014259.s001.doc]

**Table S1**: **Demographic Characteristics and Baseline Clinical Measurements of Patients with *Pneumocystis jirovecii* Pneumonia (PcP cases) and Patients with Pneumonia due to Other Causes (Controls)**

| **Characteristics** | **PcP cases (n=110)** | **Controls (n= 63)** |
| --- | --- | --- |
| ***Demographics*** | | |
| Male: N (%) **†** | 97 (88.2) | 47 (74.6) |
| Race: N (%) **†**  Caucasians  African American  Others | 58 (52.7)  35 (32.7)  16 (14.6) | 21 (33.3)  28 (44.4)  14 (22.2) |
| Age at admission (y): Median (IQR) | 40.8 (36.1-48.0) | 43.7 (38.9-48.8) |
| ***Clinical*** | | |
| CD4+ (cells/ul) less than 50 cells/ul: N (%) **††** | 68 (62.4)* | 22 (34.9) |
| Plasma HIV RNA (copies/mL: Median (IQR) **††** | 1.3x10^5^ (4.5x10^4^- 4.6x10^5^) | 3.6x10^4^ (550.5-1.4x10^5^) |
| Albumin (g/L): Median (IQR) | 3.20 (2.70-3.50) | 3.20 (2.7-3.6) |
| Prior PcP: N (%) | 17 (16) | 15 (23.8) |
| ***Pneumonia Severity*** | | |
| PO_2_ (mm Hg): Median (IQR) | 66.5 (55.0-73.0) | 65.3 (56-80) |
| Aa gradient (mm Hg): Median (IQR) | 41.0 (31.1-53.1) | 40.0 (28.0-50.0) |
| LDH (U/L): Median (IQR) **††** | 347 (270-485) | 283 (206.0-389) |
| Mechanical ventilation: N (%) | 7 (6.42) | 4 (6.4) |
| ***PcP Prophylaxis within 3 months*** | | |
| Sulfa / Sulfone N (%) **†** | 22 (20.0) | 25 (39.7) |
| ***Treatment*** | | |
| Any PcP Treatment (yes) N (%) **††** | 110 (100.0) | 42 (66.7) |
| Treatment with TMP-SMX: N (%) **††** | 82 (74.6) | 35 (55.6) |
| Steroid N (%) †† | 91 (82.7) | 26 (41.3) |
| **Serum antibody levels to MsgC1** | | |
| IgG at admission: Geometric mean (95 % CI) **†** | 18.15 (14.46-22.78) | 8.17 (6.55-10.31) |
| IgM at admission: Geometric mean (95% CI) **†** | 20.73 (16.11-26.69) | 9.16 (7.12-11.80) |

Note: N is Number of subjects; IQR: InterQuartile Endpoints. % = percent of column total. Chi-square tests were performed to test difference between PcP cases and controls for categorical data, and Wilcoxon Rank Sum test was used for categorical data

* One PcP case did not have CD4 cell count measured

**†** p < 0.05; **††** p < 0.01
