# Supplementary material for: Serum Antibody Levels to the Pneumocystis jirovecii Major Surface Glycoprotein in the Diagnosis of P. jirovecii Pneumonia in HIV+ Patients
Source: PLoS One. 2010 Dec 9;5(12):e14259. doi: 10.1371/journal.pone.0014259 (PMC3000336; doi:10.1371/journal.pone.0014259)
Supplement: Table S2 — The estimates and the associated p-value of the effects of different independent predictors on IgG and IgM antibody levels to MsgC1 in HIV-infected patients. (0.04 MB DOC) [file pone.0014259.s002.doc]

**Table S2**: **The Estimates and the Associated p-value of the Effects of Different Independent Predictors on IgG and IgM Antibody Levels to MsgC1 in HIV-Infected Patients.**

| **Model** | **Predictor** | **Effect on IgG^(a)^** | **p-value** | **Effect on IgM^(a)^** | **p-value** |
| --- | --- | --- | --- | --- | --- |
| I | PcP | 1.2 | **<0.01** | **0.86** | **0.02** |
|  | Prior PcP | 0.23 | 0.62 | -0.62 | 0.12 |
|  | CD4+ | 0.37 | 0.33 | 0.61 | 0.07 |
|  | LDH | -0.13 | **0.02** | -0.07 | 0.67 |
|  | | | | | |
| II | PcP | 0.97 | **0.03** | **0.83** | **0.04** |
|  | Prior PcP | 0.34 | 0.5 | -0.61 | 0.16 |
|  | CD4+ | 0.12 | 0.78 | 0.36 | 0.34 |
|  | PO_2_ | -0.03 | 0.83 | 0.003 | 0.99 |
|  | | | | | |
| III | PcP | 0.89 | **0.04** | **0.81** | **0.04** |
|  | Prior PcP | 0.29 | 0.53 | -0.64 | 0.12 |
|  | CD4+ | 0.40 | 0.28 | 0.57 | 0.09 |
|  | HIV Viral Load | -0.94 | 0.31 | -0.15 | 0.47 |
|  | | | | | |
| IV | PcP | 0.95 | **0.02** | 0.64 | 0.07 |
|  | Prior PcP | 0.31 | 0.50 | -0.56 | 0.15 |
|  | CD4+ | 0.41 | 0.27 | 0.62 | 0.06 |
|  | PcP prophylaxis | -0.22 | 0.59 | -0.65 | 0.11 |

Note: A linear mixed model with a random intercept was used to estimate the effect of PcP on antibody responses to MsgC1. Previous episode of PcP, CD4+ count (<50, ≥50 cells/µL), and one additional clinical variable, were modeled as fixed effects, in addition to PcP. Analyses were repeated when the additional clinical variable was replaced by a different clinical variable and all other variables remained the same.

**^(a)^** Effect = Change in log_e_ (IgG) and log_e_ (IgM) corresponding to a change in level of binary variables: PcP (yes, no), prior PcP (yes, no), CD4+ (≥ 50, < 50), PcP prophylaxis (yes, no), and one standard deviation change in continuously measured log_e_ (LDH), log_e_ (PO_2_) and log_e_ (viral load).
